# Genetic alterations associated with malignant transformation of sporadic vestibular schwannoma

**DOI:** 10.1007/s00701-021-05062-0

**Published:** 2021-11-24

**Authors:** Aril Løge Håvik, Ove Bruland, Hrvoje Miletic, Lars Poulsgaard, David Scheie, Kåre Fugleholm, Morten Lund-Johansen, Per-Morten Knappskog

**Affiliations:** 1 Department of Clinical Science, University of Bergen, Bergen, Norway; 2 Center for Medical Genetics and Molecular Medicine, Haukeland University Hospital, Bergen, Norway; 3 Department of Clinical Medicine, University of Bergen, Bergen, Norway; 4 Department of Pathology, Haukeland University Hospital, Bergen, Norway; 5 Department of Neurosurgery, Rigshospitalet, Faculty of Health and Medical Science, University of Copenhagen, Copenhagen, Denmark; 6 Department of Pathology, Rigshospitalet, Faculty of Health and Medical Science, University of Copenhagen, Copenhagen, Denmark; 7 Department of Neurosurgery, Haukeland University Hospital, Bergen, Norway

**Keywords:** Vestibular schwannoma, Malignant peripheral nerve sheath tumor, Gamma Knife Radiosurgery, Whole genome microarray, Whole exome sequencing, Malignant transformation

## Abstract

**Introduction:**

Malignant peripheral nerve sheath tumor of the vestibulocochlear nerve (VN-MPNST) is exceedingly rare and carries a poor prognosis. Little is known about its underlying genetics and in particular the process of malignant transformation. There is an ongoing debate on whether the transformation is initiated by ionizing radiation. We present here the analysis and comparison of two post-radiation VN-MPNST and one undergoing spontaneous transformation.

**Methods:**

Four tumors from three patients (radiation-naïve vestibular schwannoma before (VS) and after (VN-MPNST) malignant transformation in addition to two post-radiation VN-MPNST) were subjected to DNA whole-genome microarray and whole-exome sequencing and tumor-specific mutations were called. Mutational signatures were characterized using MuSiCa.

**Results:**

The tumor genomes were characterized predominantly by copy-number aberrations with 36–81% of the genome affected. Even the VS genome was grossly aberrated. The spontaneous malignant transformation was characterized by a near-total whole-genome doubling, disappearance of *NF2* mutation and new mutations in three cancer-related genes (*GNAQ*, *FOXO4* and *PDGFRB*). All tumors had homozygous loss of the tumor suppressor *CDKN2A*. Neither mutational signature nor copy number profile was associated with ionizing radiation.

**Conclusion:**

The VN-MPNST genome in our cases is characterized by large copy-number aberrations and homozygous deletion of *CDKN2A*. Our study demonstrates a VS with genetic alterations similar to its malignant counterpart, suggesting the existence of premalignant VS. No consistent mutational signature was associated with ionizing radiation.

## Introduction

Malignant peripheral nerve sheath tumors (MPNST) are soft tissue sarcomas arising from Schwann cells or other parts of the soft tissue surrounding peripheral nerves. Approximately 50–60% of MPNST are associated with the tumor syndrome Neurofibromatosis type 1 and approximately 10% are thought to be radiation-induced [13]. MPNST of the vestibulocochlear nerve (VN-MPNST) is exceedingly rare and carries a poor prognosis [9]. Carlson et al. estimated an incidence of 0.017 per 1 million persons per year with approximately one VN-MPNST for every 1041 vestibular schwannomas (VS) [9]. To our knowledge, only one genetic study has been performed on two cranial nerve MPNST, and hence there is a need for a better understanding of this disease [42].

Gamma Knife Radiosurgery (GKRS) is a type of ionizing radiation (IR) therapy commonly used to treat VS. There are controversies regarding whether IR might induce malignant transformation [27]. Previous studies have found unique mutational signatures attributable to radiation-induced malignancies [3, 32]. IR like gamma rays might cause all types of DNA mutations either directly by ionizing DNA molecules or indirectly by creating free radicals [33]. To assess whether GKRS induces characteristic genomic events, we compared the genome of one spontaneous VN-MPNST to two previously irradiated VN-MPNST.

To our knowledge, this is the first study on the genetics of malignant transformation of VS. To elucidate this process, we present genomic analyses of a histologically benign VS and its malignant descendant. Whole-genome DNA microarray and whole-exome sequencing were used to analyze for tumor-specific mutations.

## Materials and methods

### Patient samples

Tumor tissue and matched blood sample were collected from 3 patients without a history of NF2, who underwent suboccipital resection of unilateral presumed vestibular schwannoma (VS) at Departments of Neurosurgery at Haukeland University Hospital, Norway and Rigshospitalet, Denmark, from August 2010 to October 2016. Two patients had previously been treated with GKRS. One patient was first operated for a VS and progressed to VN-MPNST in the absence of IR as previously described [1]. Tissue was collected from all surgeries. Written informed consent was received from all patients before tissue harvesting and the study was approved by the Regional Ethical committee for medical research in Western Norway (2013/374). Tumor samples were harvested from the subcapsular part and snap frozen and stored in liquid nitrogen. All samples underwent routine histology.

### DNA extraction

For DNA extraction, tumor tissue was first disrupted using the TissueLyser (Qiagen, Hilden, Germany) and treated with protease. DNA was then extracted using the QIAamp DNA Mini Kit (Qiagen). DNA from blood was used as normal control and was extracted using QiaSymphony (Qiagen). DNA quality and quantity were evaluated with 1% SeaKem gel electrophoresis and NanoDrop (Thermo Fisher Scientific), respectively.

### Whole-genome DNA microarray

Tumor and matched lymphocyte DNA were hybridized to the CytoScan HD microarray (Affymetrix, UK) and analyzed as described [15]. Briefly, three different software were used for calling and filtering copy number aberrations (CNA) and copy number neutral runs of homozygosity (CNN-ROH); (1) Chromosome analysis suite v3.2 (ChAS, Affymetrix, UK), (2) Rawcopy [23] and (3) Nexus Copy Number (BioDiscovery, El Segundo, CA, USA). For estimating aberrant cell fraction and allele specific copy number profiles in the tumors, the Allele-Specific Copy number Analysis of Tumors 2.5.2 (ASCAT) software was used [36]. Clustering of the sample set based on CNA profiles was done with Rawcopy using the hclust R package as well as with the built-in complete linkage hierarchical clustering algorithm in Nexus Copy Number. Called variant segments from the CytoScan microarray were used for generating input for the Chromothripsis-like pattern (CTLP) scanner [39]. Identification and calculation of likelihood ratios of CTLP present in the samples were done using the website http://cgma.scu.edu.cn/CTLPScanner/ with default parameters except for the parameter of Log_2_ signal value difference between two adjacent segments that was set to 0.25.

### Whole-exome sequencing (WES)

WES and subsequent analyses on tumor-normal pairs were performed as previously described [16]. Briefly, we applied paired-end sequencing (2 × 100 bp) and aligned the sequencing data to hg19 using the Burrows-Wheeler transform, performed postprocessing of the alignments with GATK and single nucleotide variants (SNV) and indels were called using GATK and MuTect [10, 22, 24]. Annovar [37] was used for functional annotating the variants called. Filtering and prioritization of variants were done as previously described and candidates were visualized using the Integrative Genomics Viewer [30]. The Reactome pathway knowledgebase version 71 was used for pathway analysis and gene ontology (GO) annotations [17]. For visualizing and inferring the contribution of COSMIC mutational signatures in the samples, all exonic and splice site variant calls from MuTect were loaded into and analyzed in the shiny-based web application MuSiCa [12]. For comparison, we also analyzed the mutational signatures of 46 VS available from a previous study [16].

## Results

### Case reports

The first patient was operated in 2010 for a histologically benign VS (VS1), underwent spontaneous malignant transformation and was operated in 2014 for a VN-MPNST (VN-MPNST1). Biopsies from both surgeries were verified by histological examination as previously described [1]. The recurrent tumor was negative for HMB45 and melanA. The patient is still alive and doing well.

**Fig. 1:**
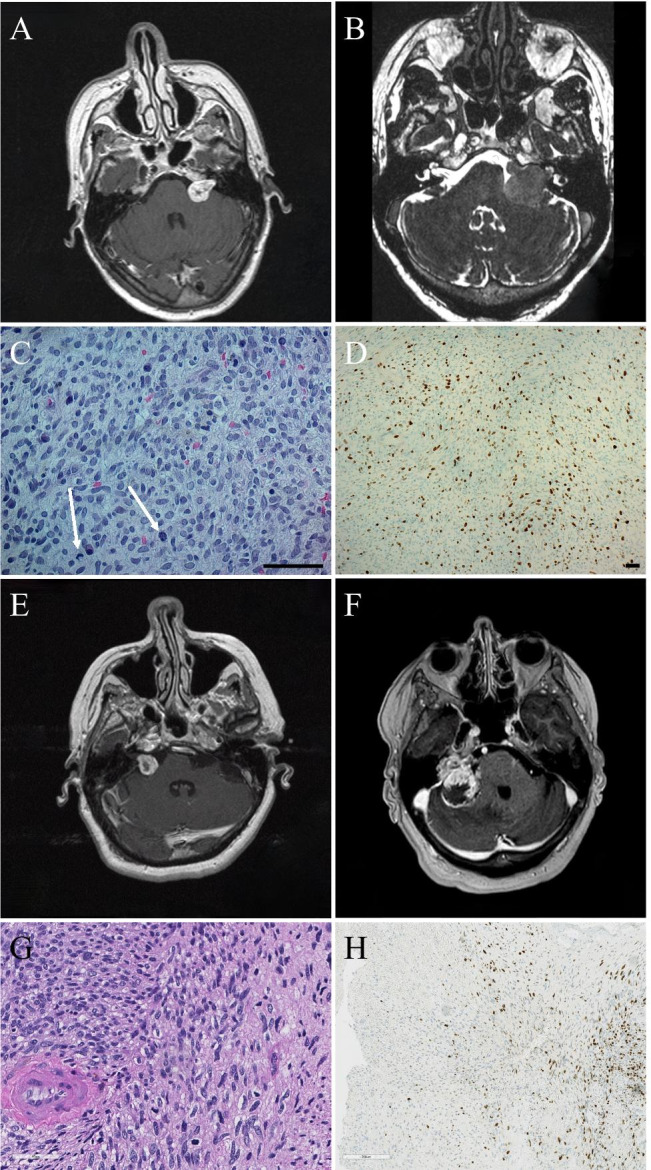
(**A–D**) VN-MPNST2: T1-weighted contrast enhanced MRI demonstrating a contrast-enhancing tumor in the left cerebellopontine angle at initial presentation (**A**) and CISS MRI demonstrating growth at recurrence (**B**). Histological examination demonstrated a hypercellular tumor with moderate nuclear pleomorphism and moderate mitotic activity (**C**, H & E, 40x, white arrows highlighting mitoses) and strong diffuse staining for Ki-67 (**D**, H & E, 10x, Ki-67). (**E–F**) VN-MPNST3: T1-weighted contrast enhanced MRI demonstrating a contrast-enhancing tumor in the right cerebellopontine angle at initial presentation (**E**) and at recurrence several years after GKRS (**F**). Histological examination demonstrated tumor tissue with high cell density and 4 mitoses per 10 HPF. Tumor cells had elongated, pleomorphic nuclei and were arranged in sheets in a fibrillary and partly myxoid matrix (**G**, H & E, 40x). Immunohistochemistry demonstrated focal positivity of tumor cells for Ki-67 (**H**, H & E, 10x, Ki-67)

The second patient, a then 63-year-old woman, presented in 1999 with an 18-month history of progressive left-sided hearing loss, and MRI demonstrated a left-sided cerebellopontine angle tumor (Fig. 1a). There was no family history or features of NF2. Follow-up scans demonstrated growth and the tumor was treated with GKRS in 2002. The tumor remained stable with a slight volume increase until 2007. In 2015, she presented with a 2-month history of increasing unsteadiness, left-sided facial numbness and weakness and diplopia. MRI demonstrated tumor growth (25 mm) and she was operated with a gross total resection through a retrosigmoid approach (Fig. 1b). Histological examination demonstrated a hypercellular tumor with moderate nuclear pleomorphism and moderate mitotic activity. The tumor was negative for S100, EMA, Desmin and Actin. Ki-67 index was around 10–30%. The tumor was diagnosed as MPNST (VN-MPNST2) (Fig. 1c, d). Ten days postoperatively, the patient was discharged to a neuro rehabilitation unit with facial nerve paralysis and glossopharyngeal nerve paresis. One year later she succumbed to the disease.

The third patient, a then 63-year-old woman, had a previous history of cystic kidney disease and renal cancer for which she had received a donor kidney and was on immunosuppressive medication. She presented in 2005 with a 3-month history of unsteadiness and a right-sided hearing loss. MRI demonstrated a contrast-enhancing tumor in the right cerebellopontine angle (Fig. 1e). The tumor demonstrated growth during follow-up and was treated with GKRS in 2007. The tumor remained stable and showed some decrease in volume up to 2014. The patient presented again in 2016 with severe unsteadiness, nausea and headache. MRI now demonstrated a 40-mm tumor with destruction of the temporal bone (Fig. 1f). On admission, she was bedridden, had difficulties swallowing and reported a weight loss of 3.4 kg over a few weeks. She was operated by retrosigmoid craniotomy. The resection, which was subtotal, was terminated due to cerebellar swelling and diffuse bleeding from the tumor and cerebellum. Postoperative CT demonstrated subarachnoid hemorrhage with intraventricular extension and hydrocephalus. External drainage was placed, and the patient transmitted to the intensive care unit. She did not regain spontaneous respiration, her consciousness gradually declined, and she died 16 days after surgery. Histological examination demonstrated tumor tissue with high cell density. Tumor cells had elongated, pleomorphic nuclei and were arranged in sheets in a fibrillary and partly myxoid matrix. Mitotic activity was observed and 4 mitoses per 10 HPF were counted. Immunohistochemistry demonstrated focal positivity of tumor cells for S100, cytokeratin AE1/AE3 and EMA, whereas GFAP was negative. Based on the histological and immunohistochemical examinations, the tumor was diagnosed as MPNST (VN-MPNST3) (Fig. 1g, h).

### Copy number aberrations

**Fig. 2:**
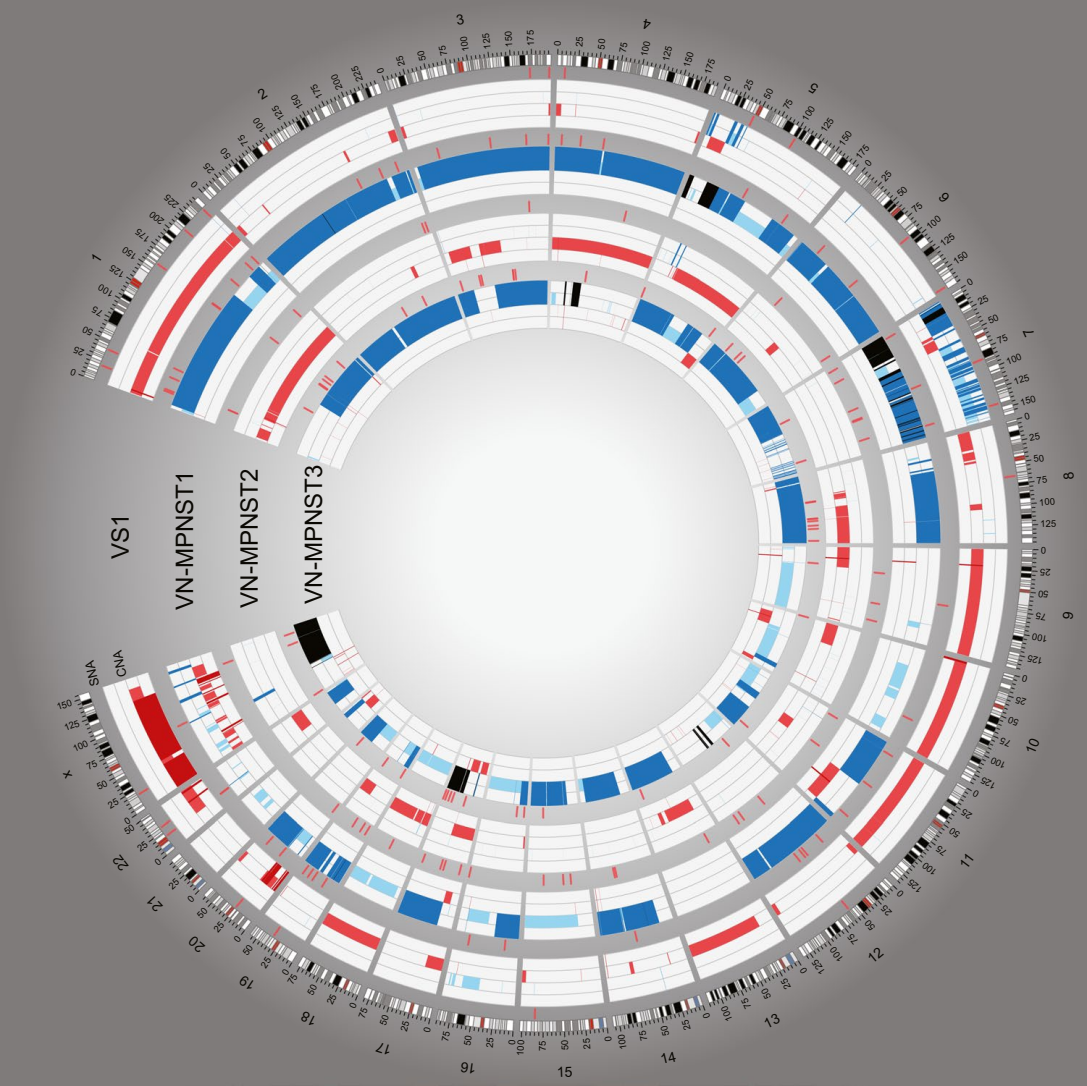
Circos plot of copy number aberrations (CNA) and single nucleotide variants (SNV) in three VN-MPNSTs and one VS, created using the Circos software [18]. The tracks from outside inwards: chromosome numbers, chromosomal position in Mb, SNV and CNA calls for four consecutive tumors and selected genes previously reported in extracranial MPNST. In the CNA histogram, high level amplifications (CN > 7), high-level gains (CN 4–7) and gain (CN = 3) is depicted in black, dark blue and light blue, respectively. Similarly, heterozygous loss and homozygous loss are depicted in light red and dark red, respectively

**Fig. 3:**
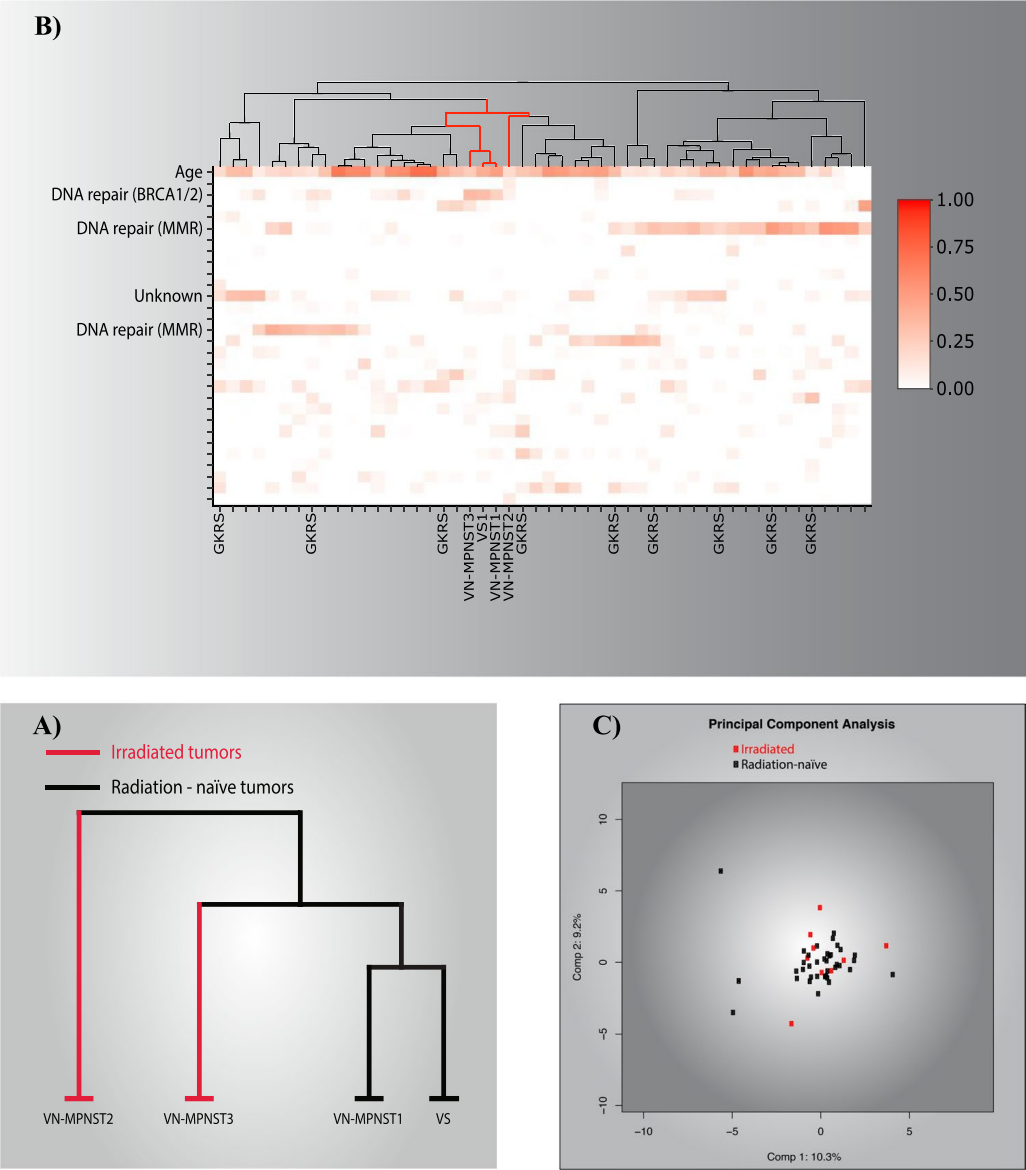
Unsupervised hierarchical clustering revealed no association between CNA profile and previous radiation exposure (**A**). A matrix depicting the relative contribution of COSMIC mutational signatures in 46 VS, one premalignant VS and 3 VN-MPNST depicted no clustering of the irradiated tumors (**B**). The columns represent the individual tumors with irradiated and malignant tumors marked along the x-axis, whereas the rows represent the 30 different mutational signatures with the signatures contributing the most marked along the y-axis. The results from hierarchical clustering of the mutational signatures are depicted on top of the matrix with malignant tumors highlighted as red lines. Principal component analysis demonstrated no association between radiation and mutational signature (**C**)

All tumors, even VS1, demonstrated grossly aberrated genomes with 36 to 81% of the genome affected by CNA (Table 1, Fig. 2). VN-MPNST 1 and 3 were hyperploid while VN-MPNST 2 and VS1 were hypoploid. No genome-wide pattern was associated with IR or survival. The tumor suppressor gene *CDKN2A*, located at 9p21.3, was affected by homozygous loss in all samples. A high-level gain of the oncogene *EGFR* was seen in VS1, VN-MPNST1 and VN-MPNST3. A heterozygous loss at 17p13, the locus for *TP53,* was seen in VS1, VN-MPNST2 and VN-MPNST3. In VN-MPNST1, CNN-ROH was seen, indicating a doubling of the locus in the progression from its benign precursor. A similar pattern was seen in chromosome 22, where *NF2* is located, with heterozygous loss in VS1 and CNN-ROH in VN-MPNST1. *NF2* was duplicated in VN-MPNST3 and harbored a heterozygous loss in VN-MPNST2. *NF1* and *SUZ12*, both located at 17q11.2, was affected by a heterozygous loss in VN-MPNST2 and VN-MPNST3, diploid in VS1 and had four copies with no allelic imbalance in VN-MPNST1. *EED* harbored a heterozygous loss in VS1 and VN-MPNST1, CNN-ROH in VN-MPNST2 and had four copies with no allelic imbalance in VN-MPNST3. Aberrant cell fraction was estimated at 31 to 81%, indicating either normal cell infiltration or different tumor clones. Unsupervised hierarchical clustering revealed no association between CNA profile and previous radiation exposure (Fig. 3a).

**Table 1:** Genetic aberrations in VN-MPNST. Key genetic findings in 1 vestibular schwannoma and 3 malignant peripheral nerve sheath tumors of the vestibulocochlear nerve. CNA, SNV and indels are called, filtered and prioritized as previously reported [15, 16]

*ID*	*Ploidy*^*a*^	*Aberrant cell fraction*^***b***^	*CNA*^***c***^	*Gain–loss-ratio*^*d*^	*Chromothripsis*^*e*^	*SNV*^*f*^	*Indels*^*g*^
VS1^*^	1.72	0.40	0.45	0.15	Chr7	20	2
VN-MPNST1^*^	3.96	0.31	0.81	15.94	Chr7	46	1
*VN-MPNST2*	1.51	0.79	0.36	0.01	None	37	4
*VN-MPNST3*	3.47	0.81	0.76	16.08	Chr7	47	2

^a^ Average ploidy across the genome

^b^ The portion of cells in the biopsy harboring copy number aberrations as estimated by ASCAT

^c^ Portion of the genome affected by a copy number aberration

^d^ The ratio of the portion of the genome affected by a gain to the portion affected by a loss

^e^ Chromosomes affected by chromothripsis as estimated by CTLPS

^f^ Number of SNVs

^g^ Number of indels

^*^Tumors from the same patient

CTLP was predicted in chromosome 7 in all but VN-MPNST2 with between 56 and 65 copy number switches across the chromosome. In VS1 and VN-MPNST1, the CTLP encompassed the whole chromosome, whereas in VN-MPNST3, 7q21.11-q36.3 was affected. The CTLP region encompassed 19, 21 and 2 Cosmic cancer census genes in VS1, VN-MPNST1 and VN-MPNST3, respectively. Two recurrent cancer genes harbored high-level gains and were included in the CTLP in all three samples, *AKAP9* and *CDK6*. Other notable cancer genes affected by CTLP in both the benign and malignant tumor from the same patient include *EGFR*, *BRAF* and *MET*, all were gains.

Regarding the progression of the VS to VN-MPNST in the absence of IR, it is evident that most of the genome has undergone a doubling. This is particularly evident from the loss of chromosome 9, 10 and 13 in VS with corresponding CNN-ROH in VN-MPNST. However, at 11q13.4-q24.3, the deletion persists in the malignancy. In the p-arm of chromosome 5, a high-level gain is evident already in VS1 with gain of even more copies in VN-MPNST1.

### Exome sequencing

The numbers of somatic SNVs and indels were similar among the VN-MPNSTs with a total number of mutations ranging from 41 to 49, whereas VS1 harbored 22 mutations. The indel-substitution ratio ranged from 2.17 to 10.81%, and no association was seen with prior radiation. No recurrent mutated gene was identified. A total of 10 mutated genes are listed as a COSMIC cancer census gene (Table 2). We found one enriched pathway using FDR cutoff < 0.05, N-Glycan biosynthesis (P-value 5.0 × 10^−5^, FDR 0.0175). All malignant tumors harbored at least one mutated gene annotated to this pathway (Table 2). We did not see any mutated genes in the Polycomb repressive complex 2, newly implied in extracranial MPNST. We did not see any mutations in *NF1* and no mutated genes in our cohort had evidence of functional interaction with *NF1*. Two genes annotated to DNA repair pathway harbored missense mutations (Table 2).

**Table 2:** Mutated genes identified through exome sequencing. All mutations reported are predicted as functional exonic mutations according to Annovar [37]

	*ID*	*Gene*	*Mutation*	*Transcript*	*cDNA*	*Protein*	*VAF*^*a*^
*Cosmic cancer gene*	VS1^*^	EPHA7	Missense	NM_001288629	c.G2338C	p.D780H	0.22
	VS1^*^	NF2	Stopgain	NM_181828	c.C235T	p.Q79X	0.12
	VN-MPNST1^*^	EPHA7	Missense	NM_001288629	c.G2338C	p.D780H	0.09
	VN-MPNST1^*^	FOXO4	Missense	NM_005938	c.C212G	p.S71C	0.20
	VN-MPNST1^*^	GNAQ	Missense	NM_002072	c.A286T	p.T96S	0.08
	VN-MPNST1^*^	PDGFRB	Missense	NM_002609	c.T1703A	p.V568E	0.15
	VN-MPNST2	CCNE1	Missense	NM_001322262	c.C554A	p.A185D	0.41
	VN-MPNST3	CDH17	Missense	NM_001144663	c.C435G	p.F145L	0.11
	VN-MPNST3	SALL4	Frameshift deletion	NM_020436.3	c.3114delT	p.K1038fs	0.54
	VN-MPNST3	TRIM24	Missense	NM_003852	c.A2744G	p.K915R	0.40
*N-Glycan biosynthesis pathway*	VN-MPNST1^*^	DPM1	Missense	NM_001317034	c.G205C	p.D69H	0.09
	VN-MPNST2	FUT8	Missense	NM_004480	c.G521A	p.R174H	0.40
	VN-MPNST2	GANAB	Missense	NM_001278193	c.C421T	p.H141Y	1.00
	VN-MPNST3	MGAT5B	Splice site	NM_144677	c.690 + 2 T > G	p.Q230_splice	0.12
	VN-MPNST3	ALG1	Stopgain	NM_001330504	c.C1009T	p.R337X	0.06
*DNA repair*	VN-MPNST2	HERC2	Missense	NM_004667	c.G8002C	p.V2668L	0.42
	VN-MPNST3	ACTR8	Missense	NM_022899	c.G19C	p.G7R	0.13

^a^ Variant allele frequency

^*^Tumors from the same patient

COSMIC mutational signature 1, the ubiquitous signature attributed to the endogenous deamination of 5-methylcytosine to thymine, contributed most to the signatures of both VN-MPNST and VS (Fig. 3b). The malignancies form a subcluster based on a relative high contribution of signature 3. This signature is associated with *BRCA1/2* mutations. We found no exonic mutations in *BRCA1/2*, but a CNA affecting either *BRCA1* or *BRCA2* was evident in all tumors. The benign tumors form two main clusters based on the presence of signature 6, which is associated with liver cancer. The tumors lacking contribution of signature 6 are further subclustered according to the presence of signature 12 and 15. The irradiated tumors do not form a distinct cluster based on mutational signature, neither through hierarchical nor principal component clustering (Fig. 3c).

Most mutations in VS1 are retained in VN-MPNST1, but with one notable exceptions: *NF2*, the commonly mutated gene in VS, harbors a stopgain mutation (NM_181828: p.Q79X) in VS1 which disappears in the malignancy. Three cancer census genes, *FOXO4* (NM_005938: p.S71C), *GNAQ* (NM_002072: p.T96S) and *PDGFRB* (NM_002609: p.V568E), acquire mutations as the tumor progresses to malignancy.

## Discussion

We demonstrate here the genetic landscape of a benign VS undergoing spontaneous malignant transformation allowing for a unique tracking of the processes accompanying this transformation. Most notably, the benign precursor harbors a grossly aberrated genome including homozygous deletion of the tumor suppressor gene *CDKN2A*, CTLP in chromosome 7 and high-level amplification at chromosome 5p. This is in stark contrast with our previous characterization of the copy number profile in sporadic VS [15], although inactivation of *NF2* is similar. Homozygous loss of *CDKN2A* has also been suggested as an initiating event in the malignant transformation of neurofibromas in NF1 patients [2, 26]. Given the low fraction of tumor cells carrying the homozygous loss, the premalignant VS might have coincided with the benign VS explaining why the diagnosis was missed with standard pathology. The loss of the *NF2* mutation in the process of malignant transformation might also indicate that two different tumor clones were present at the first surgery. CTLP is a phenomenon characterized by massive genomic rearrangements that might induce tumorigenic mutations [34]. Although CTLP have traditionally been associated with malignancies, they have also been found in premalignant lesions lending more proof to VS1 being a premalignant VS [14]. It seems that VS1, already exhibiting genomic instability, undergoes a near whole-genome doubling as well as acquires new mutations in cancer-related genes (*FOXO4*, *GNAQ*, *PDGFRB*), thereby completing the malignant transformation. Codon 209 in *GNAQ* is commonly mutated in melanocytic tumors [19]. However, we found a T96S mutation in our sample as well as negative melanocytic immunohistochemical markers, excluding VN-MPNST1 as a melanocytic tumor.

Interestingly, a global gene expression profiling performed on peripheral nerve sheath tumors identified a subset of MPNST that clustered with benign schwannomas, showed diffuse S100 reactivity and histological features indicative of schwannian differentiation [35]. Thus, it seems apparent that there exists a borderline tumor on the spectrum from benign schwannoma to MPNST. While it might not be feasible to analyze the genetics of all VS surgical specimens to detect the rare occurrence of a premalignant VS or VN-MPNST, some clinical and histologic features might indicate those in risk of malignant transformation. As seen in the previous reported cases of spontaneous transformation of benign VS, they tend to display increased Ki-67 labeling index, induce a higher symptoms load and might display uncharacteristic MRI features [5].

In agreement with previous studies on other soft tissue sarcomas, we found the tumors to harbor complex karyotypes, regions affected by CTLP and with comparably low burden of small mutations [2, 8, 25, 31]. The most notable recurrent event, homozygous loss of the tumor suppressor gene *CDKN2A*, has also been described in extracranial MPNST [6, 21, 40]. Other genes implicated in extracranial MPNST include *NF1*, *TP53* and members of the polycomb repressive complex 2 (PRC2) [7, 11, 41]. Notably, we found no small mutations in these genes. However, all tumors demonstrate heterozygous loss of either *SUZ12* or *EED*, both members of PRC2. De Raedt et al. demonstrated that reduced PRC2 dosage contributes to tumor development, hence, extending the role of PRC2 loss to intracranial MPNST [11]. The five MPNST analyzed by De Raedt et al. did not cluster together based on CNA clustering, in agreement with our results. Further, heterozygous loss of *TP53* was seen in all tumors and heterozygous loss of *NF1* was seen in two tumors. Rahrmann et al. demonstrated that *TP53* haploinsufficiency, rather than bi-allelic inactivation, may be sufficient for MPNST development [29]. Amplification of *EGFR* at 7p11.2 was found by Perrone et al. in 14/23 MPNST, and we also found high-level amplification of this region in VS1, VN-MPNST1 and VN-MPNST3 [28]. These findings, including a recent study utilizing aCGH on two VN-MPNSTs, support a similar pathogenesis in extracranial and intracranial MPNST [42].

Given the high level of genomic instability in these tumors, we sought specifically for mutated DNA repair genes. One tumor, VN-MPNST2, harbored a mutation in *HERC2*, a gene coding for an E3 ubiquitin protein ligase. This protein has been shown essential in repair of IR-induced double-strand breakage [4]. It has also been shown that *HERC2* acts as a suppressor of G-quadruplex DNA, a secondary DNA structure that triggers genomic instability, and that *HERC2* depleted cells are sensitized to the G-quadruplex stabilizers telomestatin and pyridostatin [38]. The missense mutation we found, V2668L, is predicted as deleterious by the MutationTaster algorithm. However, this variant has not been described before and it remains to be seen how it affects the protein, but it raises the possibility that it played a part in radiation-induced malignant transformation.

Two of the VN-MPNST presented here were treated primarily with GKRS, and hence, lacked histological verification of the diagnosis at the time. However, given the stable size of both tumors over a long time (9 and 12 years) until recurrence, it seems unlikely that the tumors were in fact VN-MPNST initially. A study on 80 cases of sarcoma after radiation therapy established a mean latency of 12 years (range, 3–64 years) between radiotherapy and sarcoma diagnosis, consistent with our study [20]. It also seems unlikely that a separate VN-MPNST should occur at the exact same location as a VS, given the low incidence of these tumors. Therefore, we believe that two plausible possibilities exist for the malignant transformation: (1) spontaneous malignant transformation and (2) radiation-induced malignant transformation. We found no correlation between CNA profile or mutational signature and irradiated tumors. This was also true for the 46 sVS where mutational signature was not associated with previous radiation exposure. Except for the aforementioned *HERC2* mutation in one irradiated tumor, we did not see any genetic evidence of radiation-induced malignant transformation. The main limitation of our study is the sample size. VN-MPNST are exceedingly rare, and hence, we urge other research groups with access to such tumors to collect biopsies and analyze the genome. If GKRS and other related stereotactic treatment cause malignant transformation in VS, we expect to find evidence of this in genome.

VN-MPNST is extremely rare and hence, studies on the management of these tumors are scarce. Our study demonstrates that VN-MPNST is genetically similar to extracranial MPNST. This has implications for the management of VN-MPNST, as results from clinical studies on extracranial MPNST might be extrapolated to its intracranial counterpart.

## Conclusions

VN-MPNST is a malignant tumor with grossly aberrated genome characterized by numerous CNAs and a relatively small number of small mutations, in agreement with previous studies on extracranial MPNST. Our study demonstrates a benign VS with genetic alterations similar to its malignant counterpart, suggesting the existence of premalignant VS. In the process of spontaneous malignant transformation, the tumor undergoes a near whole-genome doubling as well as acquires new mutations in cancer-related genes. No mutational signature was associated with GKRS. However, one irradiated tumor harbored a missense mutation in *HERC2*, a gene essential to DNA repair.

## Data Availability

Please contact corresponding author.

## Code availability

No code was written for this study.
